# ECG-LM: Understanding Electrocardiogram with a Large Language Model

**DOI:** 10.34133/hds.0221

**Published:** 2025-02-04

**Authors:** Kai Yang, Massimo Hong, Jiahuan Zhang, Yizhen Luo, Suyuan Zhao, Ou Zhang, Xiaomao Yu, Jiawen Zhou, Liuqing Yang, Ping Zhang, Mu Qiao, Zaiqing Nie

**Affiliations:** ^1^ Institute for AI Industry Research (AIR), Tsinghua University, Beijing, China.; ^2^ Department of Computer Science, Tsinghua University, Beijing, China.; ^3^ Beijing Tsinghua Changgung Hospital, Beijing, China.; ^4^ PharMolix Inc., Beijing, China.

## Abstract

**Background:** The electrocardiogram (ECG) is a valuable, noninvasive tool for monitoring heart-related conditions, providing critical insights. However, the interpretation of ECG data alongside patient information demands substantial medical expertise and resources. While deep learning methods help streamline this process, they often fall short in integrating patient data with ECG readings and do not provide the nuanced clinical suggestions and insights necessary for accurate diagnosis. **Methods:** Although recent advancements in multi-modal large language modeling have propelled their application scope beyond the natural language processing domain, their applicability to ECG processing remains largely unexplored, partly due to the lack of text–ECG data. To this end, we develop ECG-Language Model (ECG-LM), the first multi-modal large language model able to process natural language and understand ECG signals. The model employs a specialized ECG encoder that transforms raw ECG signals into a high-dimensional feature space, which is then aligned with the textual feature space derived from the large language model. To address the scarcity of text–ECG data, we generated text–ECG pairs by leveraging detailed ECG pattern descriptions from medical guidelines, creating a robust dataset for pre-training ECG-LM. Additionally, we fine-tune ECG-LM with public clinical conversation datasets and build an additional supervised fine-tuning dataset based on real clinical data from the hospital, aiming to provide a more comprehensive and customized user experience. **Results:** ECG-LM outperforms existing few-shot and zero-shot solutions in cardiovascular disease detection across all 3 tasks (diagnostic, rhythm, and form) while also demonstrating strong potential in ECG-related question answering. **Conclusions:** The results across various tasks demonstrate that ECG-LM effectively captures the intricate features of ECGs, showcasing its versatility in applications such as disease prediction and advanced question answering.

## Introduction

The electrocardiogram (ECG) is a widely used, noninvasive medical tool essential for detecting potential health risks and supporting home health monitoring. Medical professionals frequently rely on ECGs, combined with patient-related information such as physiological data and medical history, to make accurate diagnoses. However, despite the convenience of ECGs, their complexity and the range of possible hazards they can reveal often make it difficult for the average user, who may lack medical expertise, to interpret and understand the results.

This challenge is further intensified by the widespread need for cardiovascular monitoring, contrasted with the limited availability of high-quality medical resources. In many cases, the full potential of ECGs is not realized due to the absence of expert interpretation. For example, in large hospitals, noncardiovascular departments might require ECGs as an auxiliary diagnostic tool, yet these tests are often analyzed without considering the patient’s comprehensive health profile, leading to potential oversights. Similarly, in community hospitals or clinics, the shortage of experienced specialists can hinder detailed and accurate ECG analysis.

Outside the hospital setting, patients who use portable devices or undergo ECGs during routine checkups may receive only preliminary analyses, without the benefit of comprehensive professional interpretation. Additionally, at home, patients with cardiovascular conditions often need ongoing professional advice and health management, which may not be adequately supported by the existing ECG interpretations.

Given these scenarios, there is a pressing need for intelligent systems capable of processing ECG data in a way that integrates additional patient information and facilitates interaction with the patient. This would enable the system to gather sufficient data for accurate diagnostic recommendations and results. However, current algorithms are limited in their ability to combine basic personal health information with ECG analysis, leading to less accurate conclusions and restricting their application in real-world settings.

Supervised learning methodologies [1–3] and self-supervised learning methods [4–6] have been proposed to process ECG data [7]. While able to alleviate the need for human intervention, merely proving a result is not enough to provide the assistance that medical professionals need for accurate diagnosis.

Recently, multi-modal large language models (LLMs) [8,9] have been equipped with the ability to process both textual and nontextual information and have shown remarkable versatility, extending their application scope beyond the natural language processing domain. Their proficiency in natural language processing, coupled with the vast repository of knowledge that they encapsulate, enables them to excel in tasks ranging from text generation and image captioning to medical diagnosis and recommendation systems. However, their application in ECG processing remains largely unexplored, partly due to the insufficient availability of ECG–text data.

To this end, we develop ECG-Language Model (ECG-LM), the first multi-modal LLM that combines text and ECG signals. ECG-LM efficiently handles both modalities by aligning the feature space of a specialized ECG signal encoder with that of the LLM and achieves a comprehensive understanding of both modalities. Additionally, to address the challenge of limited text–ECG pairs during pre-training, we have designed a database expansion algorithm that leverages cardiovascular-related medical guidelines. By aligning our approach with established medical standards, we not only ensure the accuracy and reliability of the generated content but also contribute to a more comprehensive and detailed dataset. This initiative stands as a valuable addition to the field, addressing the need for well-informed and contextually relevant data in cardiovascular disease diagnostics and analysis. Results on downstream tasks show the efficacy of our approach, as it constantly outperforms other zero-shot and few-shot solutions in cardiovascular disease classification. Furthermore, we conduct fine-tuning on clinical conversation and ECG question answering (QA) to broaden the scope of ECG-LM, enabling a more versatile and personalized service.

The potential applications of ECG-LM are extensive and impactful. For users, it provides a tool that can translate complex ECG data into understandable insights, empowering them to take proactive steps in managing their heart health. By offering timely and accurate health alerts, ECG-LM can encourage users to seek medical attention promptly, potentially preventing the progression of serious conditions.

For medical professionals, ECG-LM serves as a powerful assistant, offering preliminary interpretations of ECG data and highlighting potential areas of concern. This can streamline the diagnostic process, reduce the burden on healthcare providers, and allow them to focus on more complex cases that require human expertise. Additionally, by integrating patient data and medical history, ECG-LM can offer personalized insights that support more accurate and holistic diagnoses.

Given the current shortage of medical resources and personnel, combined with long waiting times, delays in diagnosis and treatment are common, which can lead to the aggravation of symptoms. In this context, the deployment of automated intelligent systems could play a transformative role. Such systems would not only help users recognize health problems promptly and seek timely medical attention but also provide healthcare professionals with valuable insights into the patient’s condition, thereby enhancing the overall effectiveness of cardiovascular care.

Our work addresses the abovementioned challenges and aims to optimize the diagnosis and management of heart conditions, bridging the gap between sophisticated medical analysis and user-friendly communication and enhancing the overall effectiveness of cardiac care for both patients and healthcare providers.

**Fig. 1.**
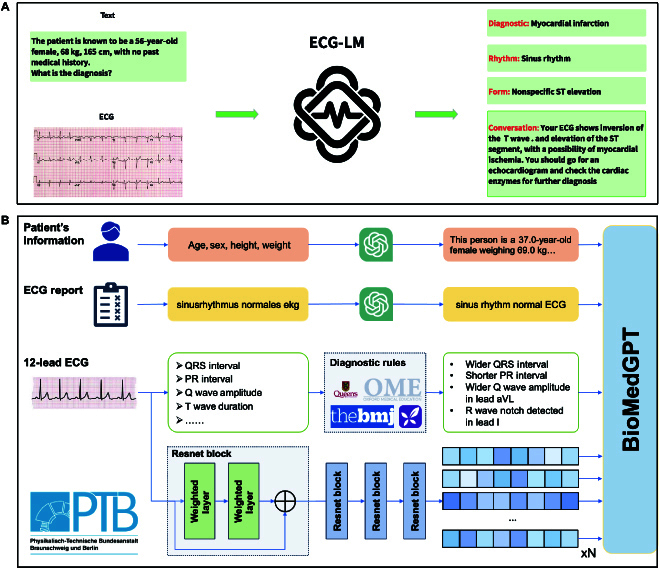
The framework of ECG-Language Model (ECG-LM). (A) The overall framework of our work, from input to output. (B) A detailed step-by-step explanation of ECG-LM, from data collection to feature engineering. Initially, we collect electrocardiogram (ECG) data, reports, and patient details from the Physikalisch-Technische Bundesanstalt-XL (PTB-XL) dataset. Non-English reports are translated using the Generative Pre-trained Transformer 3.5 (GPT-3.5) model, and numerical features are extracted and subsequently converted to textual descriptions. The data collection pipeline adheres to privacy and ethical standards to ensure accurate insights and comprehensive reports. Finally, we feed the processed data alongside the encoded ECG signal to BioMedGPT-LM-7B. aVL, augmented vector left.

The overall framework is shown in Fig. 1.

Our contributions can be summarized as follows:

• The first cross-modal LLM for text and ECG signals: ECG-LM leverages the capabilities of generative models to fuse ECG data with text while infusing medical knowledge into the LLM to enhance its intuitive understanding of ECG data features.
• A tailored data expansion algorithm: We introduce an innovative algorithm that strictly adheres to established cardiovascular medical guidelines to address the limited size of ECG–text pairs in the dataset. Furthermore, we create a supervised fine-tuning (SFT) dataset to further boost ECG-LM’s ECG-related clinical conversation skills. Our generated content has been thoroughly validated by medical professionals.
• Performance: Our model achieves state-of-the-art performance in zero-shot cardiovascular disease classification tasks, surpassing even few-shot approaches. Through fine-tuning, it outperforms larger models in electrocardiogram question answering (ECG-QA).

## Methods

### Related works

#### Electrocardiogram

In recent years, deep learning algorithms have predominantly focused on supervised diagnosis and classification of raw ECG data in an end-to-end manner. For instance, Mazomenos et al. [10] introduced a low-complexity ECG feature extraction algorithm utilizing discrete wavelet transform and Haar wavelets, extracting ECG feature points from discrete wavelet transform coefficients through modulus maxima analysis.

Hannun et al. [11] employed a deep neural network to learn from 91,232 ECG records of 53,549 patients and tested the model on ECG data of 328 distinct patients, achieving an accuracy rate that surpassed those of 78.0% of human cardiologists, particularly in the identification of atrial fibrillation and sinus rhythm. Additionally, Avanzato and Beritelli [12] proposed a novel neural architecture based on convolutional neural networks as a solution for an automatic cardiac disease diagnostic system. Moreover, an ultralightweight end-to-end ECG classification neural network (ULECGNet) [13] with minimal computational complexity is implemented on low-cost microcontrollers while achieving an overall classification accuracy of 99.1%.

These works concentrate on feature extraction from ECG data or perform relatively limited diagnostic tasks, with models exhibiting limited generalization capabilities and a lack of utilization of textual data.

#### Multi-modal LLMs

The advent of LLMs [14–16] has revolutionized the understanding and generation of natural language. Moreover, multi-modal LLMs [17–19], which aim to bridge the representation gap, facilitating effective knowledge transfer and integration between different data modalities, have displayed high adaptability and proved to be useful in many different sectors, especially in healthcare. By fostering synergy across modalities, these techniques enhance the model’s ability to extract comprehensive insights from multi-modal data, fostering advancements in areas like image captioning, audiovisual analysis, and cross-modal information retrieval. The necessity for abundant training data and the vast number of parameters of these models represent challenges for modality alignment and require specialized strategies that take into account the increased complexity and computational costs of the model.

#### Cardiovascular disease detection

Many tasks are associated with the intricate domain of ECG processing, among which the most prominent ones include diagnostic classification, rhythm classification, and form classification. Despite falling under the umbrella of classification tasks, each of these endeavors serves distinct purposes and hones in on different features of the ECG signal. All of these tasks are performed in a zero-shot manner, without requiring fine-tuning.

Diagnostic is concerned with identifying specific cardiac conditions or diseases based on patterns and abnormalities observed in the ECG signal. Once irregularities in rhythm and form are identified, diagnostic classification helps to pinpoint the specific cardiac disorder or condition. This can guide further medical interventions and treatment strategies.

Rhythm focuses on identifying the overall pattern of the cardiac cycle. It involves determining whether the heart is beating regularly or irregularly. Irregular heart rhythms, also known as arrhythmias, can have important clinical implications. Detecting and classifying the rhythm is often the first step in understanding the nature of a cardiac condition.

Form classification involves assessing the morphology or shape of individual waves and complexes in the ECG signal. This includes analyzing P waves, QRS complexes, and T waves. Abnormalities in the form of these waves and complexes can provide valuable information about specific cardiac conditions. For example, changes in the ST segment may indicate myocardial infarction.

### Medical QA

Extensive research has been conducted in the realm of QA systems within the healthcare sector, with a focus on tailoring these systems to meet specific and nuanced needs. The advent of LLMs has also substantially contributed to the refinement of these systems, not only enhancing their ability to provide precise responses to queries but also equipping them with advanced conversational capabilities. A variety of medical QA datasets are currently accessible, encompassing domains such as general medical QA [20–22], to x-ray image-based QA [23,24] and answering queries related to protein and molecule properties [8]. Notably, the ECG-QA [25] dataset has been introduced as a pioneering effort designed specifically for ECG-related QA, comprising a substantial total of 414,348 samples.

### Proposed solution

#### ECG encoder

We improve the ResNet-18 encoder [26] and add the capability to accommodate inputs of variable sizes. This modification enables the encoder to seamlessly handle any number of leads without necessitating alterations to its architecture for each scenario. Leveraging the model’s remarkable high-resolution capabilities in ECG data processing, it proves adept at unveiling abstract and concealed features within ECG signals.

The ECG encoder undergoes pre-training specifically for diagnostic superclass classification using the Physikalisch-Technische Bundesanstalt-XL (PTB-XL) dataset. During the text–ECG joint pre-training phase, we introduce a projection layer to align the output size of the encoder with the input size of the LLM. This ensures that ECG encoding functions as an individual token for the LLM.

#### Language model

Within the healthcare domain, the growing demand for high-level automated biomedical assistants has led to the development of several specialized large models tailored for biomedical tasks [27–29]. Notably, when comparing the performance of existing LLMs on PubMedQA, only 2 models outperform a human expert: MedPaLM-2 [30] and BioMedGPT-LM [8], where MedPaLM-2 is close sourced and is on a different scale, parameter-size-wise (540B compared to 7B).

As a result, we choose BioMedGPT-LM-7B as our LLM. Building upon the foundation of LLaMA2-Chat-7B, it has undergone incremental training on 4.2 million biomedical-related articles from S2ORC [31], an extensive corpus that includes numerous ECG-related publications and sources such as PubMed and PubMed Central.

#### Data collection and feature engineering

We select the PTB-XL dataset [32], a comprehensive collection encompassing a total of 21,799 clinical 12-lead, 10-s-long ECG records derived from more than 18,000 patients, as the foundational pre-training dataset for our study. Additionally, we integrated PTB-XL+ [33], a supplementary feature dataset designed to complement the former. In contrast to prior studies [34,35], which do not make use of personal information, we incorporate supplementary patient-related details, including age, sex, weight, and height, whenever available. Given that the original reports were distributed as 70.89% German, 27.9% English, and 1.21% Swedish, we translate using ChatGPT and subsequently perform manual validation to ensure linguistic and semantic consistency.

Establishing a connection between ECG signals and expert medical descriptions is instrumental in enhancing the model’s comprehension of ECG data. The PTB-XL dataset primarily consists of generalized conclusions in the form of reports, which may provide valuable insights into various cardiac conditions and abnormalities. A typical ECG signal is composed of 12 leads, which are electrodes placed on various parts of the body. Each lead captures electric signals from a different perspective, focusing on distinct aspects, views, and attributes of the heart’s activity, providing crucial information about different aspects of cardiac function and pathology. While generalized conclusions from reports offer valuable insights, they may not fully capture the complexity and nuances present in individual lead signals. Each lead signal may exhibit unique patterns and abnormalities that contribute to the overall interpretation of the ECG. Therefore, relying solely on a single generic report may limit the model’s ability to accurately interpret and analyze data comprehensively.

To enhance the model’s understanding of ECG data, it is essential to consider the multilead nature of ECG signals and incorporate information from all leads into the training process. This would allow the model to capture a broader range of cardiac abnormalities and improve its ability to interpret ECG signals accurately.

We address this limitation by extracting features computed with the 12SL tool from PTB-XL+ to enhance our dataset, specifically acquiring 12-lead-related information. We have meticulously selected features deemed valuable and, in conjunction with authoritative medical diagnostic guidelines [36–38], we assess whether the numerical values align with the normal ECG ranges for healthy individuals. We subsequently formulate templates to convert these findings to textual descriptions. We subsequently organize all the collected data into a cohesive string by employing the following template: 

This person is a <age>-year-old <gender> <sub_template>. The ECG signal is <ecg>. The ECG report indicates <report>. The anomalies in general: <general>, lead I: <descriptionI>, …, lead V6: <descriptionV6>.

As height and weight are not always available, “sub_template” is a substring that incorporates them and takes care of cases when none of the 2, 1 of the 2, or both data are available; “ecg” represents the ECG encoder’s output; “report” is the diagnosis report from PTB-XL translated to English; and “general” is a sentence that describes the global features of an individual’s ECG, while “description_lead_name” is the lead specific information retrieved through the rules.

Subsequently, we further expand our dataset using the following algorithm: Starting with an input as previously described, we randomly opt to omit information from *k* lead descriptions and embeddings, where *k* ranges from 4 to 6. In the process of dropping lead-specific information, we give priority to excluding those that contain textual information while ensuring that we do not eliminate all leads with descriptions (unless no leads had any information initially).

**Fig. 2.**
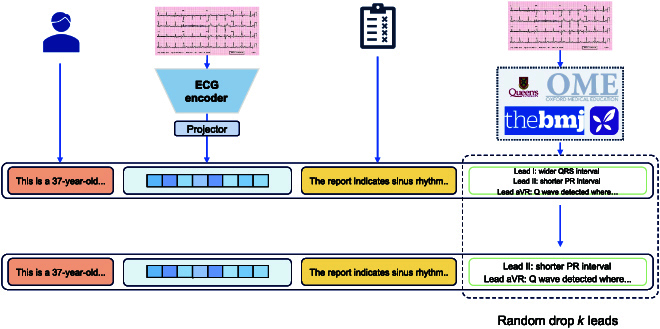
A comprehensive database construction pipeline. We expand the ECG data by randomly omitting information from a subset of leads, prioritizing those with textual data to maintain a balanced representation. We compile key metrics from authoritative guidelines to establish descriptions for the numerical features of each data entry. The process ensures that at least one lead with a description is preserved.

Utilizing the generative model’s ability to produce text in a left-to-right sequence, we establish a deeper understanding and integration between text and ECG. The database expansion pipeline is shown in Fig. 2.

For the QA task, we select ECG-QA [25]. It is the first comprehensive ECG QA dataset, built upon PTB-XL, and contains a total of 414,348 samples. It includes multiple question classes: Single-Choose (S-Choose), Single-Verify (S-Verify), Single-Query (S-Query), Comparison-Consecutive-Verify, Comparison-Consecutive-Query, Comparison-Irrelevant-Verify, and Comparison-Irrelevant-Query. Upon examination, we deem that the comparison queries lack relevance in real-world scenarios, and thus, we select only single-type questions for our work, resulting in a total of 231,536 samples.

#### Text–ECG alignment

We adapt BioMedGPT-LM to simultaneously process both text and 12-lead ECG. Throughout the entire pre-training process, the language model remains in a frozen state. This deliberate choice is made to safeguard its initial knowledge and conversational capabilities, with a paramount emphasis on conserving computational resources and minimizing associated costs. The main objective lies in training the ECG encoder to align its feature space with that of the frozen LLM.

Given an input $I=\mathrm{ecg},t_{\text{report}},t_{I},\ldots ,t_{V6}$, where *ecg* is the output of the ECG encoder, *t_report_* is the token for the general ecg report, and *t_i_* represents the tokens for the description of lead *i*, generated through medical guidelines. We compute the loss using the autoregressive function solely on tokens pertinent to the ECG report and specific leads. The function is formulated as

$$L=-\sum \text{log}\left(u_{i}|u_{j}\right)$$ (1)

where *j* < *i* and *u* can be either text tokens or ECG encoding tokens.

## Results

### Experimental settings

The ECG encoder is trained over 20 epochs with a learning rate *lr* = 1 × 10^−2^, a batch size of 64, with AdamW as an optimizer and the cross-entropy loss for classification tasks. For the text–ECG joint pre-training, the training duration is set at 20 epochs, including 4 warm-up epochs. The learning rate is initialized with 1 × 10^−4^ with a weight decay of 0*.*03 and 12 as batch size. To mitigate memory limitations, the gradient accumulation steps are configured at 8. The model is trained over a period of 1 d using 2 A100 GPUs, with 80 GB of memory, while keeping the LLM frozen throughout the process.

### Classification

To facilitate our analysis, we categorize the diverse set of labels within the dataset into 3 overarching classes: diagnostic, form, and rhythm class. Our subsequent classification efforts are centered around these consolidated superclasses. For the PTB-XL dataset, we adhere to the classification scheme outlined by Li et al. [34] for diagnostic classes, encompassing NORM (normal ECG), CD (Conduction Disturbance), MI (Myocardial Infarction), ST/T Change (alterations in the ST segment and T wave), and Hypertrophy. Rhythm labels include Sinus Rhythm (SR), Sinus Tachycardia (ST), Sinus Brachycardia (SB), Atrial Fibrillation (AFIB), and Sinus Arrhythmia (SARRH). Form labels consist of Abnormal QRS (ABQRS), Non-Diagnostic T abnormalities (NDT), Non-specific ST changes (NST_), Digitalis-effect (DIG), and Long QT-interval (LNGQT).

We format the input structure following the specifications outlined in Table 1. This structured input configuration serves as the foundation for the model’s comprehension and decision-making process across a spectrum of classification tasks.

**Table 1.** Prompt design. The prompt is given to the LLM for downstream tasks. Each task is associated with distinct labels available for selection, and we ask the language model to choose an answer from them.

Task	Prompt
Diagnostic	*This person is a {age}-year-old {gender} {sub_template}. The ECG signal is {ecg}, … Abnormalities detected in Lead I: {description_I_ }, …* Based on the given ECG signal, please identify which type (A-E) it belongs to A. Normal B. Myocardial Infarction C. ST/T Change D. Conduction Disturbance E. Hypertrophy Please identify and choose one of A, B, C, D or E as answer **ECG-LM**: {answer}
Rhythm	*This person is a {age}-year-old {gender} {sub_template}. The ECG signal is {ecg}, … Abnormalities detected in Lead I: {description_I_ }, …* Based on the given ECG signal, please identify which type (A-E) it belongs to A. Sinus Rhythm B. Sinus Tachycardia C. Sinus Brachycardia D. Atrial Fibrillation and E. Sinus Arrhythmia Please identify and choose one of A, B, C, D, or E as answer **ECG-LM**: {answer}

LLM, large language model

We assess the performance of our model using classic metrics such as accuracy, precision, recall, and F1 score. In our comparative analysis, we include multiple baseline models, spanning from few-shot to other zero-shot approaches. For the zero-shot setting, we use Multimodal ECG-Text Self-supervised pre-training (METS) [34], a contrastive learning framework that aligns the representation space of an ECG encoder with that of a bidirectional encoder representations from transformers (BERT) model. Classification is then performed by calculating the cosine similarity between the textual description and the ECG signal. Additionally, we incorporate SimCLR [39], a contrastive learning approach utilizing ResNet-18 [40] as the encoder. SimCLR employs a projection head to map the ECG embedding into a representation space governed by a contrastive loss function. For direct comparisons with other language models, we follow the approach by Yu et al. [35], replacing the original language model (ClinicalBert) in METS with LLaMA2. All few-shot experiments were conducted using 5% of the total labels. We restricted our tests on language model-based solutions to the diagnostic task.

The results presented in Table 2 demonstrate robust zero-shot capabilities. Despite being zero-shot, we markedly outperformed few-shot solutions across all metrics, showcasing the superiority of our model and the effectiveness of our alignment approach. This also emphasizes the limitations of contrastive learning, which requires a much larger batch size to be effective with LLMs. Furthermore, we note a consistently lower score on precision, recall, and F1 for tasks related to rhythm and form. This could be attributed to the imbalanced distribution of classes in the PTB-XL dataset. In the diagnostic task, a substantial portion of the ECG recordings depict healthy conditions (NORM), leading to a slightly imbalanced class distribution. Finally, we compare the zero-shot performance of our model with the supervised performance of ResNet [40] on the form classification task. While our model does not surpass the accuracy achieved by the supervised ResNet, it demonstrates a notable capability to generalize across tasks without specific training on the form classification task. This comparison underscores the trade-offs between zero-shot models, which offer versatility and broad applicability, and traditional supervised approaches, which excel when trained on large, labeled datasets. Despite not outperforming the supervised method, our model’s performance in a zero-shot context highlights its potential for applications where labeled data are scarce or unavailable.

**Table 2.** Results of zero-shot classification tasks on PTB-XL

Task	Setting	Model	Accuracy	Precision	Recall	F1
Diagnostic	Few-shot	LLaMA2-7B + METS	0.417	0.391	0.277	0.357
		LLaMA2-13B + METS	0.422	0.401	0.294	0.348
		SimCLR [39]	0.648	0.545	0.443	0.485
		METS [34]	0.3576	0.3242	0.3576	0.3178
		**ECG-LM (ours)**	**0.693**	**0.652**	**0.642**	**0.647**
Rhythm	Few-shot	SimCLR	0.660	0.446	0.471	0.456
	Zero-shot	METS	0.196	0.214	0.196	0.180
		**ECG-LM (ours)**	**0.670**	**0.509**	**0.540**	**0.524**
Form	Few-shot	SimCLR	0.697	0.516	0.565	0.549
	Zero-shot	METS	0.200	0.184	0.200	0.077
		**ECG-LM (ours)**	**0.698**	**0.573**	**0.568**	**0.570**
	Supervised	ResNet-18 [40]	0.794	0.721	0.714	0.717

METS, Multimodal ECG-Text Self-supervised pre-training.

Results from this study’s ECG-LM are bolded.

### Question answering

We follow the original train, validation, and test split defined in ECG-QA [25], and after the removal of comparison-type questions, we have 159,306 train, 31,137 validation, and 41,093 test samples. The questions are divided into 3 categories, and each of them has a different set of possible answers:

• S-Choose: given 2 options, select one of them as the answer (e.g., Which symptom does this ECG show, conduction disturbance or hypertrophy?)
• S-Query: open questions, where the objective is to retrieve the answer from a set of attributes (e.g., What kind of extrasystoles does this ECG show?)
• S-Verify: yes or no answers (e.g., Does this ECG show symptoms of nonspecific ST changes?)

We select the same baselines as in ECG-QA [25], which also suggested the use of LLMs to interpret the ECG encoder’s output. This method entails transforming predictions from the ECG encoder model into text, prompting the LLM to make decisions based on the highest probability among these converted predictions. However, performance has deteriorated after incorporating the LLM, dropping from 0.83 to 0.75, indicating that this methodology is still not fully optimized.

Additionally, Yu et al. [35] proposed a zero-shot learning method based on LLMs for ECG diagnosis, particularly for the detection of arrhythmias. This approach constructs a database filled with specific domain knowledge to guide LLMs in diagnosis without the need for training samples. While the proposed method leverages the knowledge within LLMs, it is not a multi-modal solution, as it only uses fixed textual features extracted from ECG for representation. Moreover, as we do not have access to the features and data collected by the authors, we replicate the methodology in a simplified way, by translating the ECG signal to textual descriptions based on rules we defined in the “Data collection and feature engineering” section.

The results in Table 3 show that our model consistently surpasses other LLMs across all question types. When compared to approaches not based on LLMs, we outperform every other solution in S-Verify and S-Choose while achieving the third-best performance in S-Query. This can be attributed to S-Query being essentially a retrieval task with multiple labels, and due to the nature of generative models, our pool of possible generated candidates results in being much larger compared to the fixed pool of retrieval models. However, ECG-LM still achieves the best average score, highlighting the effectiveness and potential of multi-modal LLMs. We provide an example of using ECG-LM for the QA task in Table 4.

**Table 3.** Results classified by each question type

Method	Model	Accuracy			
		S-Verify	S-Choose	S-Query	Avg
	Multi-Modal masked autoencoders for medical vision-and-language pre-training (M^3^AE) [43]	0.746	0.571	**0.410**	0.576
	Multi-Modal understanding and generation for medical images and text via vision-language pre-training (MedViLL) [44]	0.739	0.541	0.404	0.561
	Fusion Transformer	0.721	0.464	0.374	0.519
	Blind Transformer	0.677	0.310	0.240	0.409
	Deaf Transformer	0.673	0.314	0.270	0.419
LLM	gpt4 ^a^ [25]	0.710	0.481	0.357	0.549
	gpt-3.5-turbo ^a^ [25]	0.693	0.361	0.311	0.455
	text-davinci-003 ^a^ [25]	0.750	0.378	0.360	0.496
	gpt-3.5-text ^a^ [35]	0.293	0.192	0.374	0.286
	**ECG-LM (ours)**	**0.758**	**0.574**	0.399	**0.577**

S-Verify, Single-Verify; S-Choose, Single-Choose; S-Query, Single-Query; Avg, average

^a^ Tested on 10% of the test set due to application programming interface (API) usage policy.

Bold formatting indicates the best result achieved for each task.

**Table 4.** Examples of ECG-QA using ECG-LM

Task	Prompt
S-Choose	Which symptom does this ECG show, st change or myocardial infarction? Select the answer strictly from the two given options. **ECG-LM:** conduction disturbance
S-Query	What diagnostic symptoms does this ECG show, including uncertain symptoms? **ECG-LM:** non-specific st depression

ECG-QA, electrocardiogram question answering

### Ablation study

Table 5 presents the results of the ablation study conducted on ECG-LM, systematically removing one component at a time to evaluate their individual impact on overall performance. We consider 3 scenarios:

• Removal of ECG encoder pre-training: The observed performance drop underscores the significance of pre-encapsulated knowledge in the ECG encoder. This knowledge facilitates a more precise representation of ECG signals, highlighting its crucial role in enhancing model performance.
• Domain-specific knowledge: By substituting BioMedGPT-LM with LLaMA2-Chat, we illustrate that injecting relevant medical knowledge into the language model results in better understanding of domain-specific tasks and aids in enhancing its generalization capability.
• Database expansion: The most marked performance decrease is observed when omitting the data expansion step. This highlights the importance of constructing high-quality and abundant training data, which contributes to effective modality alignment and resulting in superior model performance.

**Table 5.** Ablation study on diagnostic classification

Method	Accuracy	Precision	Recall	F1
**ECG-LM**	**0.693**	**0.652**	**0.642**	**0.647**
Without ECG pre-training	0.666	0.623	0.621	0.622
ECG-LM-LLaMA2-Chat	0.670	0.627	0.635	0.631
Without expansion	0.655	0.603	0.614	0.608

Results from this study’s ECG-LM are bolded.

Even after removing individual components, ECG-LM continues to outperform the selected baselines, emphasizing the potential and effectiveness of text–ECG alignment with LLMs.

### Clinical conversation

Built on LLaMA2-7B-Chat, BioMedGPT-LM 7B already exhibits fundamental conversational skills. To enhance its proficiency in clinical discourse, we leverage the HealthCareMagic100k dataset from ChatDoctor [27], comprising 100,000 authentic doctor–patient interactions. Additionally, we built an additional SFT dataset with 2,000 doctor–patient dialogues specifically focused on ECG readings, sourced from a local hospital between 2018 and 2022, to improve its capability in ECG-related discussions. We meticulously curated the collected data to ensure that any confidential or private information, such as names, addresses, and phone numbers, was thoroughly removed.

**Fig. 3.**
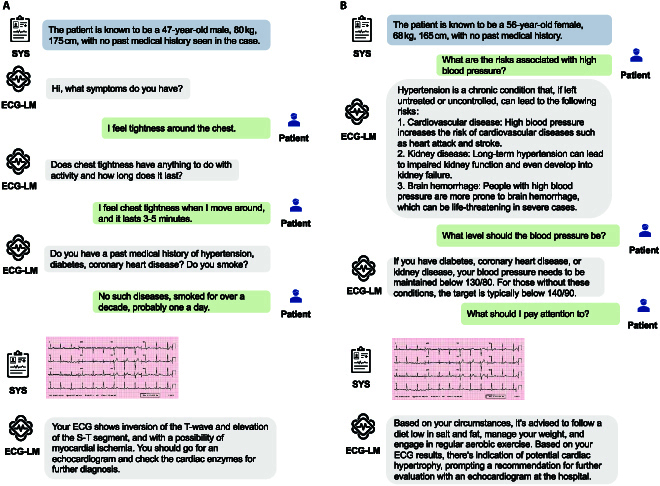
(A) The patient communicates clearly by stating their symptoms to the model. (B) The patient does not provide any information. SYS stands for system. This is information that is accessible to the model.

This process aims to augment and optimize the model’s proficiency in engaging in medical conversations, ensuring a more tailored, flexible, and contextually relevant dialogue experience. This can translate to applications where the user can initiate a conversation with a chatbot (Fig. 3), inquire about the interpretation of their ECG data, and receive instant feedback on their heart health status. The model can provide explanations about ECG patterns, offer lifestyle recommendations, and even alert users to potential health concerns based on the collected data. We offer an additional example in Table 6, where the patient’s responses include uncertainties, using phrases like “sometimes” or “Not that I know”.

**Table 6.** ECG-LM example. The answers of the patient contain uncertainties.

SYS	The patient is 52 years old, with high blood pressure, and a smoker. This is the electrocardiogram: ecg signal.
ECG-LM	The ECG seems normal, without st changes.
ECG-LM	What symptoms do you have?
Patient	I sometimes feel tightness around the chest.
ECG-LM	Have the symptoms worsened?
Patient	Not really, sometimes I feel tired.
ECG-LM	Do you have any other medical conditions?
Patient	Not that I know.
ECG-LM	You may have coronary heart disease. I recommend getting a coronary CTA scan.
Patient	What should I pay attention to?
ECG-LM	Avoid strenuous activities and overexertion. If symptoms worsen, seek medical attention immediately.

This application not only empowers individuals to actively monitor their cardiovascular health but also promotes a proactive and informed approach to well-being. The user-friendly and interactive capabilities of ECG-LM contribute to a more engaged and knowledgeable user base, fostering a comprehensive and personalized service.

## Discussion

In this work, we introduce ECG-LM, the first multi-modal LLM capable of directly processing ECG data. Unlike other approaches that convert frequency data into text, our model processes ECG embeddings generated by a specialized ECG encoder. ECG-LM was developed with the aim of improving general cardiac wellness, benefiting both patients and healthcare professionals.

Our results demonstrate that ECG-LM exhibits strong generalization capabilities, outperforming existing solutions in zero-shot and few-shot settings. While it does not yet match the performance of fully supervised models that do not leverage LLMs, this outcome aligns with our expectations. The primary goal of incorporating LLMs is to harness their conversational abilities, thereby broadening their utility and scope in the medical domain.

However, LLMs, including ECG-LM, are still prone to a critical issue: hallucination [41,42]. Even advanced models like BioMedGPT-LM-7B, based on LLaMA2-7B-Chat, are not immune to this problem. One contributing factor may be the noisy corpus used for training. As LLMs require vast amounts of data, maintaining quality at scale becomes challenging, potentially introducing noise and bias.

Moreover, our tests indicate that fully supervised methods continue to outperform LLM-based approaches in certain tasks. While we strive for optimal solutions, it is important to recognize that there is currently no way to completely eliminate hallucinations in LLMs. Therefore, we emphasize that our model is not yet ready for real-world applications and should be used only under close supervision by trained professionals.

For future research, while it is currently impossible to eliminate the issue of hallucination in LLMs, efforts can be directed toward building a more comprehensive dataset. This could involve cross-referencing the generated content with verified medical literature or databases to enhance the accuracy of the information provided by the model. Additionally, our current alignment method is relatively basic. We recommend exploring more advanced alignment techniques, such as BLIP-2 [19], to improve ECG representation learning and overall model performance.

## Conclusion

We introduce ECG-LM, a cross-modal learning model based on BioMedGPT-LM, specifically developed for advanced ECG analysis. ECG-LM effectively bridges the gap between ECG data and natural language, improving the generalization capabilities of LLMs through modality alignment. By deeply integrating text with ECG data, ECG-LM enhances its ability to understand and analyze cardiovascular features more accurately.

This research further incorporates medical guidelines and patient-specific information, using them to generate additional data and enrich the existing dataset. ECG-LM excels at extracting critical features from ECGs, identifying patterns, and offering personalized early warnings for cardiovascular conditions. Its robust generalization abilities are demonstrated through its strong performance in zero-shot tasks, including ECG diagnostics, rhythm and form classification, and QA. Moreover, ECG-LM shows substantial potential for real-world medical applications, aiming to provide comprehensive and personalized healthcare support. By enabling more accurate interpretations and timely interventions, ECG-LM aspires to bridge the gap between sophisticated ECG analysis and practical, user-friendly communication, ultimately enhancing cardiovascular care for both patients and healthcare professionals.

## Ethical Approval

This study does not involve any animal or human participants, nor does it take place in any private or protected areas.

## Data Availability

We are in the process of preparing all code and data for public release. Regarding the SFT dataset, we are collaborating with the hospital to finalize an agreement and aim to make it available as soon as possible.
